# Reminiscences of a European Trip to the International Medical Congress at Moscow, Russia, August 19 to 26, 1897, as a Delegate from the American Medical Association and the Odontological Society of Pennsylvania

**Published:** 1898-12

**Authors:** W. G. A. Bonwill

**Affiliations:** Philadelphia


### REMINISCENCES OF A EUROPEAN TRIP TO THE INTERNATIONAL MEDICAL CONGRESS AT MOSCOW, RUSSIA, AUGUST 19 TO 26, 1897, AS A DELEGATE FROM THE AMERICAN MEDICAL ASSOCIATION AND THE ODONTOLOGICAL SOCIETY OF PENNSYLVANIA.[1]

BY W. G. A. BONWILL, D.D.S., PHILADELPHIA.

(Concluded from page 712.)

We are off to "London Town" as the last of the places designated on the tour where I was expected to hold forth, and you can understand how hard at last the self-imposed burden had become, which had not been fully considered, when the statement was made by the journals that clinics would be held at certain cities. Up to the hour for leaving for home (on the grandest steamer afloat) not one hour's indisposition had been experienced; not even in Rome, in the hottest and most risky season of the year.

One week now in London. I was met at the cars and at once conducted to a hotel near to the old Tomes offices, where every minute of time was laid out for me in entertainments, talks, clinics, and visiting the dental schools, which were now in full working order. The stay was too short, preventing me from meeting many good men who were out of town on their vacation, and I had to forego seeing my friend Charles Tomes, and his former assistant, Mr. Baldwin. However, Mr. Baldwin left his flats at my disposal, to go and come at pleasure, and his associates left nothing undone to make the time spent a thing long to be remembered.

I had not been long in the hotel before a delegation came in to give me the programme for the week.

My old friend Cunningham, of Oxford, was not slow in putting in his appearance, to talk up his Manual Training School, and have me not neglect his pet scheme, the education of boys in the art of knowing what to do with their fingers as well as their hands. While upon this subject let me say that he has had a great deal of courage to engage in such an undertaking; one which can never do him credit and justice, as it requires too much of his life and robs him of too many hours from his practice and from his rural home amidst the old educational buildings of Oxford, where he lives a bachelor's life with his devoted mother. I told him it would prove a thankless pro-

---

[1] Read before the Odontological Society of Pennsylvania, March 12, 1898.

cedure and leave him quite penniless and chagrined to find at last that it had not realized his ideal. It is a work for the city to do, not for one man. While the object is a commendable one, yet it is not a wise one, for he can fill in his time and expend his energies to far better advantage to himself and the world by adhering strictly to his practice. I hope he will see it as I do, and relinquish it before he finds himself losing practice in endeavoring to teach boys their A-B-C's before entering a dental school. His attention to me was brotherly and lovely, and the hours I spent in his office and at his picturesque home were among the most pleasant of my stay.

He gave me an insight into his methods and general practice, which evidenced his efforts to save human teeth, combined with a boundless love for the work. It was in the right direction, yet nowhere in Europe could I find the contour system developed as in America.

His method of immediately placing in a normal arch any tooth, however irregular, I could not regard as justifiable. It can be done, but it is a question with me, as I fairly said to him, whether it could not be accomplished by better methods.

His work in this line was principally at the hospital, where the class of patients had no means for correction of irregularities, and this was his principal plea for the practice. It is my opinion that it would have been better for them to have been given artificial teeth at once at the expense of the hospital.

He saw the barriers in his way, and I feel sure he will hardly carry out the plan. It seemed sufficient for him to know that he could make it a success in some cases, and with that as an experiment he should rest satisfied, and acknowledge that prudence dictates its abandonment even in the children that are found among hospital cases.

I had hoped to meet personally both Mr. Charles Tomes and Mr. Baldwin, his successor, but they could not wait any longer for me, their hour of vacation having arrived. Particularly was I desirous of meeting Mr. Tomes, as he had taken such an antipathy to my views against evolution, and had taken the trouble to secure the opinion of a celebrated mathematician in regard to my drawings. His reply was, "Why this man is trying to square the circle." Mr. Tomes had never heard me explain my discovery of the anatomical laws of the human jaw. He knew nothing of it himself, and had no right to place any estimate upon it, *pro* or *con*, but should have awaited my demonstration.

Aside from my own deductions, he could have easily seen the laws underlying not only all the art of prosthesis, but of general anatomy, and he should have embodied the result in his dental anatomy, and let the evolution part of it go for another occasion. I will remind him just here that he will one day give me as much or more credit for this discovery than anything I have done, and his regard for me will only be the greater. He has said too many good things about my work to attempt to cast me into ridicule and kill it by sarcasm, as he has tried to do in his anatomy.

Mr. Baldwin was of great assistance to me in 1889, on my visit to the First International Dental Congress in Paris, where he accompanied me, and was my patient and assistant. I found him one of the best all-round men I met, and I regret he was not in London while I was there.

As I before stated, he placed his flat and servants at my disposal, but I could not enjoy the luxury, as every minute was consumed in London. His two companions in practice made up well for his absence. Both elegantly entertained me individually at dinner, and gave me attention every hour of my stay.

It was my intention to call upon Dr. J. Leon Williams, but I had him mixed up in my addresses with another Dr. (Lloyd) Williams, and the very limited time before sailing prevented my seeing him. An invitation had been sent me before leaving home to speak and clinic before the British Dental Association that met at Dublin, but it was impossible for me to be present.

I never fail to see the Coffin family, as their gentle demeanor and kind attentions to me in 1881, on my first visit to London, and again in 1889, was most conducive to my pleasure. While the head of the house has gone, yet they hold together, and I found them as lovely as ever to each other and to me. The Coffin boys as well as Dr. Bonnell were with me much. Mr. Walter, from indisposition, had given up his fine practice, but is again by degrees getting into the harness. He is a man well posted on most all subjects, and is a splendid conversationalist. It was refreshing to me to meet him. An evening was spent with him and the family out of London, where love and harmony reigns.

Ash & Sons and the Dental Manufacturing Company were visited, and their stock and manufactory examined. Surely no men could take more pride in their work, and their constant endeavor seems to be to have the dental world have the advantages of their means and facilities in the production of everything needed in practice.

Several American dentists in London gave me attention, and joined in one of the dinners given me, and were very kind. So much time was consumed on the social side every evening, and visiting of dental schools with talks and clinics during the day, that half a day was all I had for outside pleasure; but I gave it up freely that I might mingle with men who are generous gentlemen and make no distinction between nationalities.

Amalgam is used here by the ton. The copper amalgam must go out of use when it is known that failures do not come alone from the material used in filling, but from the shaping of cavities, how far to cut, and, above all, in knowing how to pack in amalgam to prevent leakage and recurrence of decay.

Mr. Tomes and his former assistant, Mr. Baldwin, are using my method of packing amalgam under bibulous paper, following it with dry chips of the alloy to absorb all mercury.

An American dentist at one of the dinners given to me in London was extolling cataphoresis in a case of removal of a live pulp in a second inferior bicuspid. The whole operation consumed about thirty-five minutes. I asked him how long it took to produce obtunding? This was rather stunning to him. Thirty minutes alone for the current left him five minutes for filling the pulp-canal and gutta-percha for the crown. I gave my experience of years before in the same line by the simple current alone, and the operation was completed immediately, with no wasting of half an hour and no rubber dam; and now I remove a living pulp without anything, simply holding the breath, without rapid respiration, unless the pulp be inflamed. This takes about thirty seconds,—made in three moves: first, drilling or exposing fully the pulp; second, passing broach to the extremity of the root; third, twisting once the broach and complete extirpation; three separate inhalations with an intermission of a few moments to exhaust the lungs each time.

To consume half an hour in obtunding the pulp is ridiculous. A minute quantity of arsenic would have given no severe pain if an application had first been made to subdue inflammation. It does very well for dentists who are not busy to fool their patients in this manner, but it is wrong practice.

I met at the same dinner party another American dentist, who practised in London and elsewhere, who had told me when I was in Europe in 1889 that he had doubled his practice in dollars in the past two years. He volunteered to tell me the secret of such a marked success. He said, "Since I became familiar with gold crowns, I no longer take the trouble to fill teeth with gold or amalgam, if at all difficult, such as I once did with your electric mallet, and resulted in such a satisfaction to me. I now cut off all such cases posterior to the cuspids. This pleases the class of patients coming to me, and I get double pay for it."

Such a practice is an explanation of why so many all-gold crowns and gold bridges are placed on teeth, not only in Europe but in America, and is a blot upon the dental profession, and is, in fact, doing away with the highest art in dentistry,—the filling with gold, amalgam, and other materials. The dollar rules the hour with dentists as well as the rest of the world. But this craze for crowns and bridges has swept over the dental world, and the man who will not place on gold caps and insert bridges by mutilating human living teeth must go by the board, for he is considered not up to a high professional standard. Besides, it has so far superseded the practice of sectional plates, held by clasps, that there has been a falling away from this commendable practice. Any ignoramus can stick on by cement a poorly fitting gold cap to a mutilated tooth, sharpened to let it go on loosely. In all such work I observed, both here and at home, that the laws of articulation are comparatively lost sight of.

To record my personal experiences with the many with whom I came in contact would make a volume.

I visited the schools and watched closely the work done by students in the clinics, and found as much to commend in the teaching by demonstrations and operations as anywhere in America. At Guy's Hospital one thing pleased me above all else seen in the schools. At each chair there was an individual wash-bowl with an ample supply of towels, so that no student could have an excuse for dirty hands and instruments. This is an innovation worthy of commendation, and well calculated—better than all the germ teaching—to make them careful and thoroughly clean operators. This always brings success, and comes nearer to making dentists gentlemen. This was in strong contrast to many colleges which I had visited in America, where only one and never more than half a dozen bowls were found in the clinic rooms. I can, however, say more for these schools now, for in the past few years new buildings have been reared, with more complete equipment. Were I an investigator of bacteria I would make search to discover why the pulp-canals are not invaded to the apical foramen and cut out for us, instead of our having to use drills and sulphuric acid in nearly every case treated.

In one thing I saw no advance, here or anywhere in Europe, and which, in my judgment, would secure more success in the art of filling with any and all materials. Until greater contours (what I call "plus contours") are made, no approximal fillings are safe from subsequent caries. This can only be done by taking more time to gain space on interproximal walls, by wedging with gutta-percha for weeks and months, and then, when amalgam is more thoroughly understood, and ability is acquired to pack it, and when gold is used less on all teeth posterior to the cuspids, will there be an advance in this line, more teeth saved, and fewer of the detestable gold crowns and bridges used.

Until the system of charging a given or a fixed sum for every operation, simple or compound, is thrown aside, no man can do his duty. The temptation to put in as many operations as possible during the day prevents the possibility of charging by the character of the operation and time consumed thereon. More teeth could be saved, would men but rise above this prevailing custom, and have the courage to charge for amalgam what it is worth when well done.

I have so often been asked, "Bonwill, do you get paid for all the temporary gutta-percha fillings you put in?" My reply to all is, "Yes; my patients even pay me for my thoughts (my advice), as it saves them from many operations." The system of requiring all students who enter the schools in this country to be fully taught in mechanical work before they matriculate is worthy the highest praise. This should always be done. If the student is incapable in this, then he should not be allowed to pass a collegiate course. Let the student attempt to get clear of the dirty work of the laboratory, and he will be worthless. The students in London are from a higher class than most of those in America, and better educated, and stand a better chance to rise socially. Another important matter: we should have access to the offices of all, and see what is being done to private patients, or special clinics should be given by the best men, where all those in full practice can go to measure their qualifications.

As I remarked before, the dental world here must more fully recognize that, instead of so much talk over the science of bacteriology and how to combat the germs that are said to be the active agents in the decay of human teeth, if they would put their wits to work and look with the eye instead of the microscope, they would find that the mechanical relations existing in the mouth would give a philosophy of action and almost complete prevention of recurrence of caries, after filling with gold, amalgam, or oxyphosphate, or even gutta-percha. More time should elapse between the first excavations made and the completion of the final filling, that a plus condition of the approximal surfaces should be had, and then doing away with the rubber dam in the preparation of cavities at the first sitting. This gives full opportunity to cut up high under the gum-border and removing all superfluous soft tissue, and allow time for separation and healing of the parts and the restoration of the alveolus. To this we must add the almost complete abandonment of gold in any shape, and learn how to use a proper amalgam, and how to make these plus contours; and, I must further add, to learn to charge for amalgam such prices as are commanded for gold fillings,—proportionately, I mean. To this one other item which cannot be overlooked or failure will surely come,—to never fill upon the approximal surfaces without cutting through upon the occlusal and trimming all weak walls that mastication is sure to break away, and when both approximal surfaces are involved to make a triple compound filling. Do not fear the cavities cannot be shaped to hold the filling. The cavities are abandoned too soon, and gold caps substituted where amalgam would preserve for years. Then use all porcelain crowns.

Too much gold, not cutting away the walls far enough, and too little interproximal space! These three points fully observed will entail upon the germ theory a rightful oblivion. Not forgetting that, germs or no germs, proper filling, and previous preparation, there are some teeth doomed to go to the wall, from innate constitutional want of molecular vitality to hold the atoms together; and yet these can be saved many years by the practice herein stated. Unless we carry out the golden rule, or (I will say) the *amalgam* rule, and do as we would have others do to us, we cannot succeed with any practice, and this can only be done by men who are naturally adapted in every sense for this highest of all arts upon the human body. *More than all else that results in failure is the work of incompetent operators.*

To say more of my trip would consume too much time, and try the patience of readers who know Bonwill's failing to exhaust whatever he undertakes. A volume could well be written of what occurred. But let this suffice. I trust all here and abroad, who may happen to see this article, will credit me with having so conducted my talks and clinics given as to honor dentistry everywhere. These have not come from an "American dentist," but from a cosmopolitan, willing to impart whatever he had gained in a life of forty-four years' service in the dental profession.

For mistakes or wrong impressions given to those whom he met he will be pardoned, as all has been said to help on our work for humanity. The only regret is that more could not have been done in the short time allotted at each place, and see more of actual practice at the chair. Yet no one was ever shown more of this, nor could men have acted more generously in every respect.

The tour was well ended in the last dinner given me, where both English and American dentists were present. Here the stern realities of the office were set aside, and the social came in to make one feel that without such minglings we would lose much of the amenities of professional intercourse, and the world outside would be a meaningless name and "life not worth the living."

The hour having arrived for my departure, I was escorted by my young friend, Dr. Schelling, to the cars, and I could say adieu to all Europe with a thankful heart for the pleasant stay and the treatment vouchsafed me, and that my life was yet spared and in excellent health.

The highest honor I felt I received was on reaching home. To be let alone, as I had always been, to follow out my own inclinations and ideas. Not a man came to see me to say, "Bonwill, I am glad that you received such honors and represented us so well;" and at my society meeting even the president and secretary left the room without speaking a word of comfort. But it remained for the Chicago profession to finish up the grand tour by giving me a good time at their clinics, February 21 and 22, and the general and special attention during my stay there.

I trust this trip will teach me how many good fellows there are in the world, and that I have more friends than foes, and, after all, that home was worth having, life worth living, and *dentistry worth practising.*

